# Isotopic niche reflects stress-induced variability in physiological status

**DOI:** 10.1098/rsos.171398

**Published:** 2018-02-21

**Authors:** Agnes M. L. Karlson, Martin Reutgard, Andrius Garbaras, Elena Gorokhova

**Affiliations:** 1 Department of Environmental Science and Analytical Chemistry, Stockholm University, Svante Arrhenius väg 20, 106 91 Stockholm, Sweden; 2 Department of Ecology, Environment and Plant Science, Stockholm University, Svante Arrhenius väg 20, 106 91 Stockholm, Sweden; 3 Mass Spectrometry Laboratory, Centre for Physical Science and Technology, Savanoriu 231, 02300 Vilnius, Lithuania

**Keywords:** trophic niche, stable isotopes, growth and body condition, stress, environmental contaminants, nutritional status

## Abstract

The isotopic niche has become an established concept in trophic ecology. However, the assumptions behind this approach have rarely been evaluated. Evidence is accumulating that physiological stress can affect both magnitude and inter-individual variability of the isotopic signature in consumers via alterations in metabolic pathways. We hypothesized that stress factors (inadequate nutrition, parasite infestations, and exposure to toxic substances or varying oxygen conditions) might lead to suboptimal physiological performance and altered stable isotope signatures. The latter can be misinterpreted as alterations in isotopic niche. This hypothesis was tested by inducing physiological stress in the deposit-feeding amphipod *Monoporeia affinis* exposed to either different feeding regimes or contaminated sediments. In the amphipods, we measured body condition indices or reproductive output to assess growth status and δ^13^C and δ^15^N values to derive isotope niche metrics. As hypothesized, greater isotopic niche estimates were derived for the stressed animals compared to the control groups. Moreover, the δ^15^N values were influenced by body size, reproductive status and parasite infestations, while δ^13^C values were influenced by body size, oxygen conditions and survival. Using regression analysis with isotope composition and growth variables as predictors, we were able to discriminate between the amphipods exposed to nutritionally or chemically stressful conditions and those in the control groups. Thus, interpretation of isotopic niche can be confounded by natural or anthropogenic stressors that may induce an apparent change in isotopic niche. These findings stress the importance of including measures of growth and health status when evaluating stable isotope data in food web studies.

## 1. Introduction

Isotopic niche analysis is a subtle way of quantifying change in the trophic structure of communities and populations [1–3]. Ratios of ^15^N to ^14^N (expressed as δ^15^N) exhibit stepwise enrichment with trophic transfer serving to derive a trophic position (TP), whereas ratios of ^13^C to ^12^C (δ^13^C) change little with trophic transfer and, thus, can be used to determine ultimate sources of dietary carbon. By quantifying inter-individual variation in bivariate δ^13^C–δ^15^N space, information on trophic structure and niche breadth of the population is assessed [1,2]. In the last decade, this approach has become popular, enabling a comeback of the niche concept in ecology [4]. Moreover, stable isotope analysis (SIA) has become a standard tool in ecotoxicology, particularly in bioaccumulation and biomagnification studies, because it facilitates TP estimates in a food web [5,6]. Recently, ecotoxicologists have also started using isotopic niche metrics (isotopic diversity indices *sensu* Brind'Amour and Dubois [7]) to investigate effects of contaminant exposure on the food web structure and functioning [8,9].

There are some uncertainties, however, associated with the use of isotopes in ecology and, particularly, in stress ecology and ecotoxicology [10,11]. Exposure to contaminants and other stress factors (e.g. nutritionally unbalanced food, temperature and parasite infestations) may change isotope fractionation via metabolic pathways involving synthesis and turnover of major biomolecules [12–15]. Isotope niche analysis relies on the assumption that variability in diet–consumer fractionation is negligible among individuals. However, an increased variance in individual responses is frequently observed under suboptimal conditions, which is related to phenotypic and genetic differences among coexisting individuals [16–18]. In such conditions, the intra-population variability in metabolic capacity to withstand stress would probably manifest as increased variability in fractionation and, thus, increased isotopic niche size. In line with that, individual growth rate was found to be a significant driver of Layman niche metrics, including niche size, in mysids feeding on the same diet [19]. Hence, interpretation of isotopic niche and TP estimates may be confounded by environmental stress. From the environmental assessment perspective, the challenge is to determine whether a sampled population is experiencing a stress. Hence, variability in the isotope composition could potentially be used as a diagnostic tool, particularly when combined with measures of physiological status.

In this study, we conducted two experiments to test effects of food depletion and chemical stress on the δ^13^C and δ^15^N values in the deposit-feeding amphipod *Monoporeia affinis.* Growth, reproduction and survival in this species are dependent on the input of diatoms during spring bloom, a high-quality food source [20–22]. As a result of food limitation, preferential breakdown and elimination of light isotopes via excretion and respiration may occur and lead to enriched δ^15^N and δ^13^C values in starved animals, e.g. [12,19,23]. Moreover, as alterations in amino acid and fatty acid metabolism are expected during food limitation [24], the isotope fractionation can be further affected. Also, amphipods have a high lipid content [25], and utilization of these storage lipids, which are depleted in δ^13^C relative to bulk, would elevate the bulk δ^13^C signal [26]. However, when the δ^13^C data are lipid-corrected, this elevation should not occur [27]. A chemical stress may result in feeding inhibition but may also promote catabolic processes; both effects would ultimately lead to greater δ^15^N and δ^13^C enrichment [13,14], unless the exposure causes a severe metabolic depression, in which case the response in δ-values might not be apparent.

We hypothesized that a chemically or nutritionally stressed population would have a larger isotopic niche than a non-stressed population when their diet is held constant. As a proxy for chronic toxicity, we used embryo viability, because bioassays and field studies have shown that exposure of *M. affinis* to contaminated sediment induces embryo aberrations, e.g. [28–30]. Using individual isotope signatures in concert with measurements of reproductive performance (Experiment 1) or body condition and growth (Experiment 2), we addressed three questions: (i) Is there a relationship between isotope composition and growth or reproductive performance? (ii) Do stressed amphipods show higher intra-population variability in isotope composition (i.e. higher values for all Layman niche metrics)? (iii) Can intra-population variability in isotope composition and growth/reproductive performance be used to identify physiological stress?

## 2. Material and methods

### 2.1. Experiment 1: effects of contaminant exposure on reproductive output and isotope composition

#### 2.1.1. Experimental sediments and animals

Sediment was collected in August 2010 at two sites in the northern Baltic Sea, outside the city of Piteå. The first site is relatively pristine, located approximately 30 km from the coast (monitoring station N6–2; 64.9158° N, 22.146° E; designated as *reference site*), and the other site is located in the coastal area that receives an effluent from a pulp mill plant (station 12B; 64.20°45′ N, 21.39°28′ E; designated as *contaminated site*). Using a grab sampler, we collected the top 2 cm of the surface sediment, removed macrofauna using a 0.5 mm sieve and stored the sediments in darkness at 4°C until the experiment and chemical analyses.

The details on the chemical analyses of the sediments and the results are reported elsewhere [31]; see also electronic supplementary material, table S1 for a short summary. In brief, the concentrations of polychlorinated biphenyls, heavy metals, chlorophenols and polycyclic aromatic hydrocarbons were considerably higher at the contaminated site compared to the reference site. The concentrations in the reference sediment were below the critical levels defined by Swedish EPA [32]; see also [31].

At the beginning of October, *M. affinis* were collected in a coastal area with no known local sources of pollution (near the Askö field station in the northern Baltic proper, 58.889° N, 17.649° E). The amphipods were kept in a 50 l container at 4°C with continuous water flow until the start of the experiment at the end of October. Oxygen concentration was monitored.

#### 2.1.2. Experimental set-up: chemical exposure

The amphipods were exposed to the experimental sediments (reference versus contaminated) in 2 l mesocosms (Erlenmeyer flasks, seven replicates per treatment) for three months. The flasks were covered with green plastic film to simulate natural light conditions at 20–30 m depth; see Reutgard and Furuhagen [33] for details. Air and water temperatures were kept at 4°C. To each mesocosm, 400 ml of sediment and 1 l of brackish water were added 1–2 days before the start of the experiment, when 25 females with visible gonads and 25 males were introduced. Mesocosms received a continuous flow of brackish water (3.6 ml min^−1^). To prevent food deficiency, 245 mg of Tetraphyll®, a commercial fish food commonly used as a positive control in feeding experiments [33,34], was added to each mesocosm after 42 days of exposure; this amount corresponds to 14 g C m^−2^ input of high-quality organic matter, which would represent high-level feeding conditions [34]. The incubation was terminated in mid-January, after 88–95 days of exposure. Temperature, oxygen levels, water flow, salinity and pH were monitored twice a week throughout the experiment.

#### 2.1.3. Analysis of reproductive performance and isotope composition

At the termination of the experiment, all females were weighed (wet weight including the brood) and examined for visible parasite infestation with nematodes; the infestation status was used as a categorical variable, i.e. present or absent. Thereafter, embryos were removed from all gravid females (*n* = 94 in the reference and *n* = 113 in the contaminated treatment) and classified as normal or aberrant after examination under stereomicroscope; the diagnostics followed the system of Sundelin and Eriksson [35]; see electronic supplementary material, table S2 for details. In total, we analysed 2899 and 2862 embryos from the control and exposed treatments, respectively. For each female, we determined fecundity (F; number of embryos per female) and number of viable embryos (VEs; number of embryos without aberrations per female). These variables were used in statistical analyses; the percentage of the VEs per brood (VE%) was also calculated.

After the embryo removal, the females were snap-frozen and stored at −80°C until preparation for SIA. To provide a start value of the isotopic niche metrics in each treatment as well as the individual weight values at the start of the experiment, 15 individuals originating from three additional mesocosms and acclimatized to the experimental conditions during five weeks were also used for SIA. The animals were dried at 60°C, weighed and transferred to tin capsules for SIA; these individuals are referred to as *initials for reference sediment* and *initials for contaminated sediment* hereafter.

### 2.2. Experiment 2: effects of feeding regime on growth performance and isotope composition

#### 2.2.1. Experimental sediments and animals

In May 2006, surface sediment and *M. affinis* were collected near the Askö field station. Amphipods and sediment were treated as in Experiment 1 until the start of the experiment (temperature at these depths below the sub-thermocline is 4–5° all year round). Spring bloom material consisting of diatoms (95% of the total phytoplankton biovolume, dominated by *Achnanthes taeniata*) was collected from Himmerfjärden Bay (see Nascimento *et al*. [34] for details).

#### 2.2.2. Experimental set-up: food type and quality

*Monoporeia affinis* were exposed to different sources of organic matter added to the sediment; four treatments were applied: (i) spring bloom material dominated by diatoms and representing naturally occurring high-quality food; (ii) Tetraphyll, also representing a high-quality food; (iii) lignin (Curan 100: a refractory by-product from the paper industry, obtained from Holmen Paper, www.holmen.com), representing a poor food source for these animals [34,36]; and (iv) ambient sediment, serving as a reference and representing a low-nutrition treatment. The elemental composition, amino acid content and fatty acid profiles (data extracted from [34]) of the added organic matter sources confirmed that diatoms and Tetraphyll were indeed superior foods, with much higher contents of aminoacids, nitrogen, phosphorous and higher proportion of essential fatty acids than bulk sediment and lignin (electronic supplementary material, table S4).

Each 2 l mesocosm (*n* = 5) with a surface area of 50 cm^2^ contained 500 ml of sediment collected after the spring bloom, 450 ml of brackish filtered water and 24 individuals of subadult *M. affinis* (individual body mass 1.1 ± 0.3 mg dw); this population density corresponds to 2400 ind m^−2^, resembling population densities in the field [37]. To each mesocosm, 9 g C m^−2^ of organic matter was added, corresponding to a spring or summer bloom sedimentation in the area [38].

The experiment was terminated after five weeks. At the termination, oxygen profiles were measured in each mesocosm (three profiles per mesocosm) with a Clark-type microelectrode (Unisense OX50, Aarhus, Denmark) at ambient temperature, and the weighted mean oxygen penetration depth in the sediment was calculated as described in Karlson *et al*. [39]. *Monoporeia affinis* are sensitive to low oxygen concentrations, which may reduce swimming and feeding activities [40]. Amphipod survival in each mesocosm was recorded, and all recovered individuals were snap-frozen.

#### 2.2.3. Analyses of body condition, growth performance and isotope composition

For each individual, isotope composition (δ^13^C and δ^15^N) and physiological status were determined (*n* = 50–99 individuals; table 1). To assess the physiological status, we used (i) individual body mass (dry weight), a measure of somatic growth; (ii) the RNA : DNA ratio, a proxy for protein synthetic capacity [41], as shown for various animals, including *M. affinis* [42]; and (iii) the carbon to nitrogen (C : N) ratio, a proxy for lipid content in *M. affinis* [43]. The rationale for using these variables is elaborated in electronic supplementary material, table S3. To focus on changes in muscle (i.e. protein-rich) tissue of the experimental animals, nucleic acids were measured using the sixth pereiopod dissected from five individuals per mesocosm. Nucleic acid measurements were performed with the RiboGreen assay according to Gorokhova and Kyle [44]. The rest of the body was dried at 60°C, weighed, packed in tin capsules and shipped to UC Davis Stable Isotope Facility, California, for analysis of δ^13^C and δ^15^N and elemental content (C and N). Animals sampled at the start of the experiment (after experimental acclimatization of five weeks) were also included in SIA, C : N and body mass (*n* = 15), and the RNA : DNA ratio (*n* = 9) analyses to estimate the niche size and growth status at the start of the incubation.

**Table 1.** Summary of the statistical analyses used to test Hypotheses 1–3. To remove the isotope baseline effect between the treatments, anomalies (individual values normalized to the group mean; denoted by a) were used in H2; see Material and methods for details. *Exposure* refers to the contaminated sediment treatment (in comparison to the reference sediment; categorical factor 0 or 1) and *parasite* denotes parasite-infested individuals (categorical factor 0 or 1). *Feeding regime* includes 4 levels (treatments): reference, lignin, diatoms and Tetraphyll. In both experiments initial individuals were included as an additional group for pairwise niche metric comparisons.

hypothesis	dependent	independent variables	method	*n*	results
experiment 1					
H1a: Higher intra-population variability in physiological and isotope responses in chemically stressed amphipods	F, body mass, VE%, δ^15^N_,_ δ^13^C	reference versus exposure	Levene's test	15–52	table 2, figure 1*a*
	ED	exposure + parasite +mortality	LMM	95	table 3, figure 1*a*
	Layman niche metrics, SEA_B_	initials versus reference versus exposure versus parasite	Bayesian inference	15–52	figure 3*a* and electronic supplementary material, figure S3–S8, table 4
H2a: Elevated δ^15^N and δ^13^C values are linked to deteriorated physiology of amphipods in toxic exposure	δ^15^N_a_, δ^13^C_a_	body mass + parasite + F + VE% + mortality	LM	95	table 5
H3a: Chemically stressed populations can be discriminated using a combination of physiological and isotopic measurements	exposure (reference/exposure)	δ^15^N_a_+ δ^13^C_a_+ ED + F + VE%	logistic GLM	75	table 6
experiment 2					
H1b: Higher intra-population variability in physiological and isotope responses in nutritionally stressed amphipods	RNA : DNA, C : N, body mass, δ^15^N_,_ δ^13^C	reference versus feeding regime	Levene's test	10–15	table 2, figure 1*b*
	ED	feeding regime + oxygen + mortality	LMM	50	table 3, figure 1*b*
	Layman niche metrics, SEA_B_	initials versus reference versus diatom versus Tetraphyll versus lignin	Bayesian inference	10–15	figure 3*b* and electronic supplementary material, figure S9–S14, table 4
H2b: Elevated δ^15^N and depleted δ^13^C values are related to slow growth of amphipods in nutritionally poor environments	δ^15^N_a_, δ^13^C_a_	body mass^4^ + RNA : DNA + C : N+ oxygen + mortality	LM	50	table 5
H3b: Nutritionally stressed populations can be discriminated using a combination of physiological and isotopic measurements	feeding regime (high/low quality)	δ^15^N_a_+ δ^13^C_a_ + ED + body mass + RNA : DNA + C : N	logistic GLM		table 6

### 2.3. Stable isotope analyses

#### 2.3.1. Stable isotope calculations

The C and N isotope ratios are expressed in the ‰ notation, using the equation

2.1 $$\delta (‰)=\left(\left[\frac{R_{\mathrm{sample}}}{R_{\mathrm{standard}}}\right]-1\right)\times 10^{3},$$

where *R* is the ratio between the heavy and light isotopes (^13^C : ^12^C or ^15^N : ^14^N). The stable isotope ratio, denoted by δ, is defined as the deviation in ‰ from an international reference standard (Vienna Pee Dee Belemnite for C, and atmospheric nitrogen gas for N). Samples were run in continuous flow with a standard deviation of less than 0.2‰ (UC Davis (Experiment 2) and Mass Spectrometry Laboratory, Vilnius [13] (Experiment 1).

The δ^13^C values of the amphipods was corrected for lipid content (C : N ratio) according to Post *et al*. [45] because all animals had a C : N ratio greater than 3.6, the recommended threshold for normalization.

Euclidean distance (ED) in isotopic space between the individual δ^15^N and δ^13^C values to the centroid value for the population (treatment) was calculated as

2.2 $$\mathrm{ED}=\surd ({\left(\delta^{13}C_{a}\right)}^{2}+{\left(\delta^{15}N_{a}\right)}^{2}).$$

The δ^13^C and δ^15^N values were normalized by calculating anomalies (denoted by *a* in equation (2.2)) from the population mean within treatments to remove the effect of different isotope baselines in the different sediments (Experiment 1) or feeding treatments (Experiment 2). ED was used as a predictor in some of the statistical models for hypothesis testing when individual endpoints were used as the response variable, described further in §2.4.2.

#### 2.3.2. Isotope niche analyses

Isotope niche metrics [2] were calculated for lipid-corrected SIA data in each experimental treatment and the initial animals using the SIBER computational code included in the package SIAR [3,46] (accessed 21 June 2017) in R v. 3.4.0 [47]. The Layman metrics represent different aspects of trophic diversity. Distance to centroid (CD) is the average ED (equation (2.2)) for all individuals to the δ^13^C–δ^15^N centroid, providing a measure of the average degree of trophic diversity. Convex hull area is the total area (TA) encompassed by all points on a δ^13^C–δ^15^N bi-plot; it emphasizes the role of individuals in overall dispersion within isotopic niche space. The mean nearest neighbour distance (MNND) describes how individuals are distributed relative to one another within a dietary niche space of the population, with lower values indicating less divergence. The standard deviation of this metric, SDNND, gives information on the evenness of dietary divergence. Finally, the δ^15^N range (NR) quantifies trophic length of the population, while the δ^13^C range (CR) indicates multiple basal resources and a potential for niche diversification. The standard ellipse area (SEAc) [3] provides information about the core aspects of a population's niche; it is less sensitive to outliers and small or uneven sample size than the convex hull area [3,48].

### 2.4. Hypotheses and statistical models

An overview of the statistical methods used to test the hypotheses is found in table 1. The rationale for the models is as follows.

#### 2.4.1. Treatment effects on amphipod physiology

In Experiment 1, we expected that amphipods exposed to contaminants and those infested with parasites would have lower biomass, fecundity and embryo viability (VE%) compared to the animals in the reference treatment [28–31]. Similarly, in Experiment 2, amphipods supplied with high-quality food (diatoms and Tetraphyll) were expected to have higher growth and body condition than those in the lignin and reference sediment treatments as well as the initial animals [21,34]. To test these expectations, individual-based data (body mass, fecundity, embryo viability, C : N ratio, RNA : DNA ratio and ED) were used as response variables in linear or generalized linear mixed-effect models (LMMs or GLMMs; table 1). The restricted maximum likelihood algorithm was used to estimate variance components; *z*-statistics (for *Fecundity* that follows a Poisson distribution) or *t*-statistics (for all other variables that were normally distributed) were used to test fixed effects. In all LMMs, the fixed factor was *treatment* (control and contaminated sediments in Experiment 1, and food treatments in Experiment 2), with the random factor *mesocosm* nested under *treatment*. When evaluating the fixed effect, the random effect was always retained in the model, because the mesocosms were part of the experimental design to capture environmental variation. In Experiment 1, the females with parasite infestation comprised about 25% in both treatments (table 2). As the infestation was similar between the treatments, it is likely that females were carrying the nematode already at the start of the experiment (as suggested for microsporidian infestations in *M. affinis,* [50]). Parasites may influence sensitivity to contaminants [50] and this variable was, therefore, used as an additional fixed factor (*parasites*, crossed with *treatment*). Amphipod survival was used as a covariate when evaluating effects on body mass, fecundity and embryo viability to control for possible size-selective mortality effects (table 1). In Experiment 2, oxygen penetration depth and amphipod mortality were used as covariates when evaluating effects on body mass, body condition and RNA : DNA to control for suboptimal feeding conditions [40] and size-selective mortality (table 1). Analyses were carried out using the lme4 and lmeTEST packages [51,52] (accessed 20 April 2017) in R studio v. 3.1.1 [53].

**Table 2.** Summary of survival, physiological metrics and isotope composition in initial amphipods and from Experiments 1 and 2. For fecundity, median values and lower and upper quantiles (in brackets) are given; otherwise, values are mean ± s.d. Asterisks denote non-homogeneity of variances as compared to the reference treatment in each experiment. See Material and methods for details on the various statistical models.

	experiment 1			experiment 2				
endpoints	initials	reference	exposure	initials	reference	lignin	diatoms	Tetraphyll
survival (%)	NA	63 ± 11	74 ± 13	NA	47 ± 12	41 ± 12	44 ± 12	44 ± 15
parasite infestation in females (%)	—	27	25	—	—	—	—	—
fecundity (embryo/female)	—	29 (21.5–39)	23 (18–33)*	—	—	—	—	—
embryo viability (%)	—	92.0 ± 0.16	71.5 ± 0.36*	—	—	—	—	—
individual body mass (dry weight, mg)	2.49 ± 0.74^a,b^	2.78 ± 0.88^a^	2.51 ± 0.77^a,^*	1.05 ± 0.35	1.19 ± 0.43	1.18 ± 0.57^ms^	1.43 ± 0.38	1.49 ± 0.62
RNA : DNA ratio	—	—	—	1.54 ± 0.64	1.45 ± 0.08	1.44 ± 0.16	1.85 ± 0.15	1.84 ± 0.21
C : N ratio	4.57 ± 0.54	4.55 ± 0.13	4.58 ± 0.25	5.93 ± 0.74	5.63 ± 0.91	5.45 ± 1.29	6.67 ± 0.42*	6.16 ± 0.50*
δ^15^N amphipod	10.11 ± 0.32	9.97 ± 0.28	10.01 ± 0.41*	8.32 ± 1.16	9.48 ± 1.48	10.95 ± 2.00	10.24 ± 0.88^ms^	9.87 ± 0.90^ms^
δ^13^C amphipod^c^	−19.26 ± 0.31	−18.89 ± 0.38	−18.92 ± 0.56*	−20.55 ± 1.45	−20.36 ± 1.50	−20.62 ± 1.81	−19.37 ± 1.02	−18.97 ± 0.76*

^a^Body mass including brood, converted to dry weight from wet weight according to Rumohr *et al*. [49].

^b^Average value all initials (see electronic supplementary material, table S5).

^c^Values corrected for the C : N ratio according to Post *et al*. [45].

^ms^Marginal significance, *p* < 0.06, *p < 0.05.

#### 2.4.2. Hypothesis 1: higher intra-population variability in physiological and isotope responses in stressed amphipods

As a result of the commonly observed higher variability in physiological responses under stress [14,16,19], we expected an increase in intra-population variability of physiological and δ-values in the malnourished and contaminant-exposed amphipods compared to amphipods from the reference treatments (Hypothesis 1). To test whether physiological and isotope data (RNA : DNA, body mass, C : N, F, VE%, δ^15^N, δ^13^C) had higher variance under stress compared to the optimal conditions (i.e. the reference in Experiment 1 and the diatom and Tetraphyll treatments in Experiment 2), Levene's test for homogeneity of variance was used. Moreover, we tested whether amphipods supplied with high-quality food in non-stressful conditions would have less variable isotope signatures reflected by their lower ED values using LMMs as described above. Testing effects of the crossed terms (*parasites* and *treatment*) on ED was not possible due to the lower sample size for the isotope than for the reproductive datasets. To visualize separation of the treatment groups in each experiment, projection to latent structures by means of partial least squares (PLS bagplot) was used. We expected lower values for all isotope niche metrics during optimal conditions than under stressed conditions induced by suboptimal feeding regimes, contaminant exposure or parasites. The SEA values were compared using Bayesian inference as described in Jackson *et al*. [3]; these are referred to as SEA_B_. To compare the other Layman metrics among the treatment groups that differed in sample size (*n* = 10–54), they were bootstrapped (*n* = 2000) based on their sample size [54]. Probabilistic distributions representing 50%, 75% and 95% credible intervals of mean estimates are presented as density plots for each metric and treatment in both experiments.

#### 2.4.3. Hypothesis 2: isotopic response to physiological variability

Generalized linear models (GLMs) were used to evaluate the driving forces of variability in the isotope data. We expected that fast-growing amphipods had a higher uptake of C from added food than slow-growing amphipods [13]. We tested the effect of relative weight gain from the initial weight and treatment and their interaction on the absolute change in δ^13^C (and δ^15^N) using a GLM with normal error structure and log link function in R studio v. 3.1.1 [53]. When significant interactions were found, the treatment effects were tested separately.

Our next hypothesis (Hypothesis 2, table 1) was that higher δ^15^N fractionation and greater negative δ^13^C fractionation in growing animals experiencing nutritionally poor environment [19] would result in a negative relationship between growth and δ^15^N values and a positive relationship between growth and δ^13^C values. Under toxic exposure, in non-growing adults, the physiological responses indicating deteriorating health status would be linked to elevated δ^15^N and δ^13^C values [13,14]. The relationships between physiological and isotope variables were evaluated using linear models with normal error structure and identity or log link function. As the absolute differences in isotope signatures between the treatments would be largely driven by the isotopic signature of the sediment or added food, which also might have become more enriched during the experimental duration [55], we normalized the data to represent the change that occurred during the exposure. To remove the effect of different isotope baselines between sediments and added organic matter, anomalies of the δ^13^C and δ^15^N values were calculated as described (§2.3.1). In Experiment 1, δ^13^C and δ^15^N values were related to body mass, reproductive performance (F and VE%), parasite infestation [15] and amphipod mortality (table 1). In Experiment 2, δ^13^C and δ^15^N values were related to body mass, C : N ratio, RNA : DNA ratio and weighted mean oxygen penetration depth as well as amphipod mortality (table 1). Mortality was included as a predictor since high mortality could potentially result in scavenging and hence enriched isotope values of surviving individuals, whereas low oxygen penetration depth was expected to reduce swimming activity and affect respiration-driven changes in δ^13^C [40]. The Akaike information criterion (AIC) was used to select the most parsimonious model.

#### 2.4.4. Hypothesis 3: combining physiological and isotopic measurements to identify stressed populations

To evaluate whether the ED values, the normalized δ^13^C and δ^15^N values and reproductive or growth performance of the amphipods can be used to discriminate between the stressed (contaminant exposure or malnutrition) and non-stressed populations, logistic models were applied (Hypothesis 3, table 1). We used *treatment* (Experiment 1: contaminated versus reference sediment; Experiment 2: high-quality versus low-quality food) as a binomial response variable and the individual ED values, the δ^13^C and δ^15^N anomalies and growth status measured either as reproductive performance (Experiment 1: F, %VE) or growth indices (Experiment 2: RNA : DNA ratio, C : N ratio, individual body mass) as predictors (table 1). The C : N ratio within a treatment was normalized to *z*-scores (zero mean, unit variance) to improve normality of the residuals. The models were tested using a binomial error structure, and the AIC score as the selection criterion. Odds ratios of the logistic regressions were used as a measure of associations between the treatment and the predictors. Prediction accuracy of the models was evaluated using the receiver operating characteristic (ROC) to calculate the area under curve (AUC). The sensitivity (i.e. true positives, or the proportion of cases correctly identified by the model as being unaffected by stress) and the specificity (e.g. true negatives, the proportion of cases correctly identified by the model as experiencing stress) were extracted from the ROC curves [56]. The logistic regressions and subsequent model prediction procedures were performed in RStudio v. 3.1.1 using packages ROCR and AUC [57,58] (accessed 18 May 2017). A single outlier (see electronic supplementary material, figure S1*b*) was removed from the dataset and replaced with the treatment mean prior to all calculations and statistical analyses described above. In all models, residuals were examined to confirm that the selected error distribution and link function were appropriate.

## 3. Results

### 3.1. Experiment 1: effects of contaminated sediment on reproductive performance and isotope composition

The median survival rate was 70% and did not differ significantly between the treatments (Mann–Whitney *U* test, *Z* = −1.54, *p* = 0.12, *n* = 7; table 2). Body mass did not change significantly during the experimental duration for reference or contaminant-exposed amphipods compared to their initial size (table 2, electronic supplementary material, table S5). As expected, body mass, fecundity (F) and embryo viability (VE%) were lower in the contaminant-exposed amphipods compared to the reference animals (electronic supplementary material, table S6). Embryo viability in the reference sediment was in the range of what is normally found at reference sites in the Baltic Sea [35]. There was a significant interaction effect between sediment contamination and parasite infestation on F; with lower F and VE% values observed only in the infested females from the controls.

**Figure 1.**
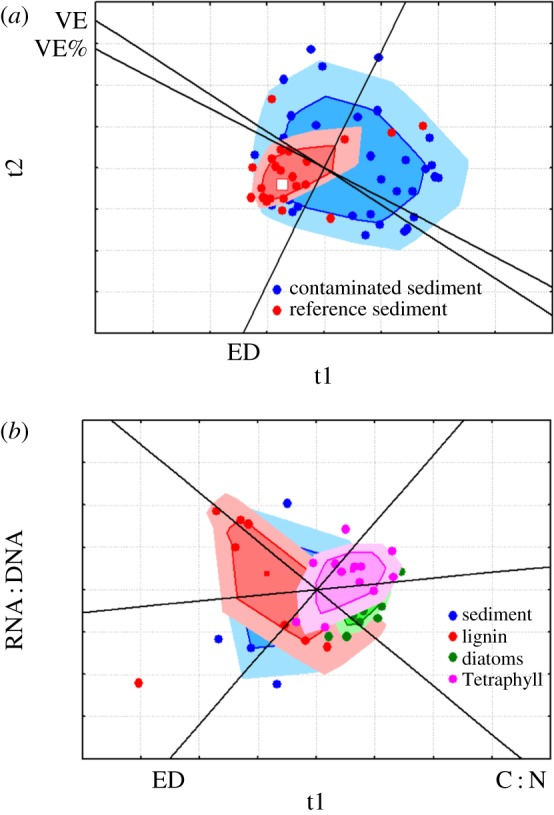
PLS bagplots for the models with *treatment* as a categorical response variable for (*a*) Experiment 1: reference versus contaminated sediment with isotope data (ED), VEs per female (VE) and proportion of VEs (VE%) as explanatory variables; and (*b*) Experiment 2: the four feeding treatments with ED, growth status (RNA : DNA) and body condition (C : N) as explanatory variables. Each point on the score plot for the first two latent variables (t1 and t2) represents an individual *M. affinis* sample projected in the bivariate space, and loading scores provide the correlation between the original variables and the component variables. The structure of the multivariate point cloud is represented by the shaded areas in the bagplot, analogous to a box-and-whiskers plot but also visualizing the spread, correlation, skewness and tails of the data. The dark area corresponds to 50% of the dataset, and the light area is a fence augmented by a default factor of 1.5. Sample points outside the shaded areas are outliers.

The variances in amphipod body mass, F, VE%, δ^13^C and δ^15^N (Levene's test; table 2) and ED to centroid for individual isotope signatures (ED, table 3) were significantly higher in the contaminated treatment. The variability in the reproductive and isotope variables in both treatments is visualized in a PLS bagplot (figure 1*a*).

**Table 3.** Most parsimonious linear mixed-effect models (LMMs) testing treatment effects on ED of isotope signatures in Experiments 1 and 2. As a treatment variable, exposures to contaminants in Experiment 1 and organic matter supplements (diatoms, lignin and Tetraphyll) in Experiment 2 were used; reference treatment was set as the reference in both experiments.

variable	estimate	s.e.	*t*-value	*p*-value
experiment 1				
exposure	0.0830	0.0830	3.481	0.0008
survival	−0.0236	0.0120	−1.973	0.0515
experiment 2				
diatoms	−1.1055	0.3818	−2.895	0.0113
Tetraphyll	−1.4533	0.3892	−3.734	0.0020
lignin	−0.3025	0.3956	−0.765	0.4553

### 3.2. Experiment 1: isotope niche metrics in reference and contaminant-exposed amphipods

**Figure 2.**
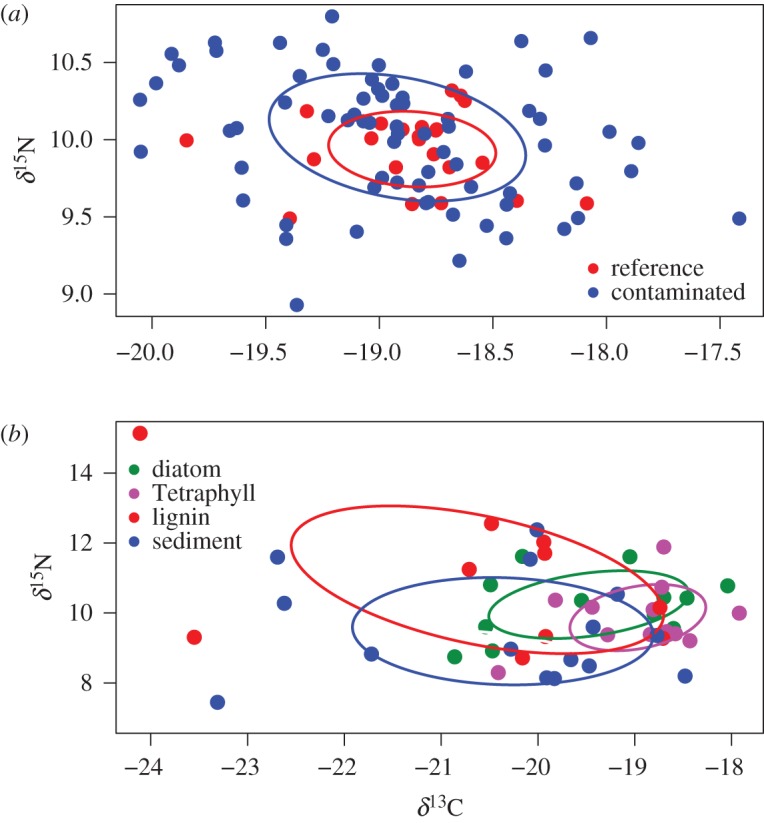
Stable isotope bi-plots illustrating the isotopic niche of the *M. affinis* in Experiments 1 (*a*) and 2 (*b*). The lines enclose the standard ellipse area (SEAc) and illustrate the relative difference in size between ellipses (the exact position of ellipses are not interesting here). Sediment in (*b*) refers to reference treatment. See figure 3 for credible intervals of SEAc in each treatment and experiment, and electronic supplementary material, figures S3–S14 for density plots of each of the Layman metrics.

**Figure 3.**
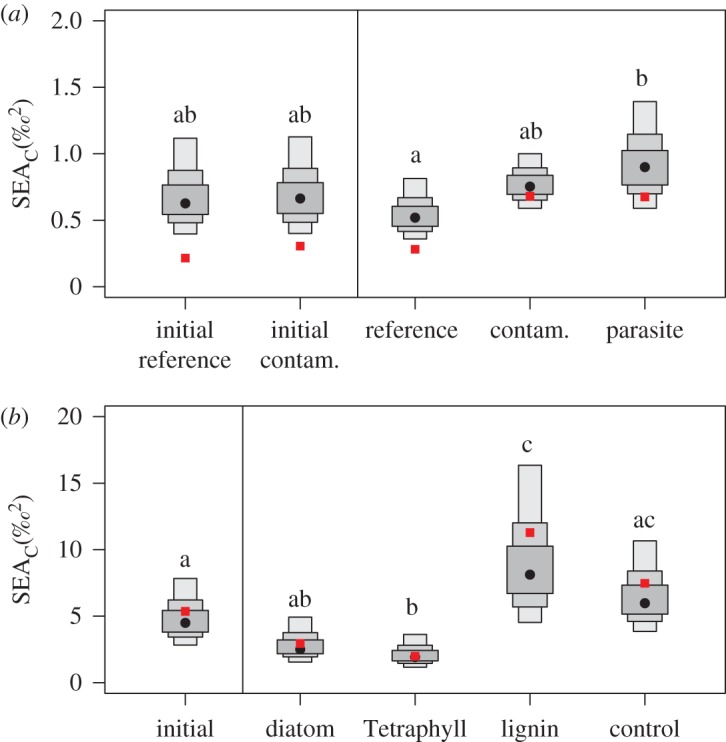
Density plots of standard ellipse area (SEA) for each treatment and for the initial amphipods (to the left of the vertical line). Boxed areas indicate the SEA_B_ (black dot) with Bayesian 50, 75 and 95% credible intervals and the red square represents SEAc (see text for details). Parasitized amphipods from the contaminated treatment in Experiment 1 are shown as a separate group here. Control in Experiment 2 refers to reference sediment only, with no organic matter added. Common letters denote no significant difference according to Bayesian inference (*p* > 0.05).

Isotope niche metrics for amphipods sampled in the beginning of the experiment were similar between the treatments (table 4). At the end of the experiment, amphipods in the reference treatment had unchanged or moderately higher values for CR, TA and SEAc and SDNND compared to the initials, while values for NR, CD and MNND were unchanged or lower (table 4, electronic supplementary material, figures S3–S8). Contaminant-exposed amphipods had several-fold higher values for all isotope metrics except for MNND and SDNND compared to the initials (Hypothesis 1, table 4, electronic supplementary material, figures S3–S8). Moreover, compared to the reference treatment, all isotope metrics except for MNND and SDNND were several-fold higher in the exposure (Hypothesis 1, table 4, figure 2*a* and electronic supplementary material, figures S3–S8). Also, the parasite-infested individuals from the exposed group had higher values for all metrics compared to those in the reference sediment (table 4, figure 2). The SEA_B_ was higher in the contaminant-exposed amphipods than in the reference sediment (*p* = 0.08), and the parasite-infested individuals had significantly higher SEA_B_ than the reference (*p* < 0.05, figure 3*a*).

**Table 4.** Isotope niche metrics for amphipods in Experiments 1 and 2. The data from Experiment 1 are shown for the starting group (initial) and for the reference and the contaminant exposure at the termination of the experiment (final); the parasite-infested individuals are shown separately from the exposed group. The data from Experiment 2 are shown for the starting group (initial) and for the four feeding regimes at the termination of the experiment (final). All Layman metrics were bootstrapped (*n* = 2000, denoted with b) based on the minimum sample size in the dataset to allow more robust comparison of the mean values. SEA is corrected for sample size according to Jackson *et al*. [3]; see also figure 2, and denoted SEAc. Values in brackets show the change in per cent relative to the initials for the respective treatments. See density plots in electronic supplementary material, figures S3–S8 (Experiment 1) and electronic supplementary material, figures S9–S14 (Experiment 2) for each of the niche metrics.

	experiment 1					experiment 2				
	initial		final			initial	final			
niche metrics	reference (*n* = 15)	exposure (*n* = 15)	reference (*n* = 23)	exposure (*n* = 52)	parasitized (*n* = 21)	(*n* = 15)	reference (*n* = 15)	diatoms (*n* = 12)	Tetraphyll (*n* = 12)	lignin (*n* = 10)
NR_b_	1	0.8	0.8 (−20)	1.6 (90)	1.4 (70)	4.2	4.9 (20)	2.0 (−50)	2.4 (−40)	6.4 (50)
CR_b_	0.7	1	1.5 (110)	2.4 (150)	2.0 (100)	4.6	4.8 (10)	2.8 (−30)	2.5 (−50)	5.4 (20)
TA_b_	0.4	0.5	0.7 (90)	2.6 (420)	1.4 (190)	13.6	9.6 (−30)	4.2 (−70)	3.1 (−70)	19.3 (40)
SEAc	0.2	0.3	0.3 (30)	0.7 (120)	0.7 (120)	5.6	7.5 (40)	3.0 (−40)	2.0 (−60)	11.3 (110)
CD_b_	0.4	0.4	0.3 (0)	0.6 (50)	0.6 (60)	1.2	2.0 (60)	1.0 (−20)	0.8 (−30)	1.6 (30)
MNND_b_	0.1	0.1	0.1 (0)	0.1 (0)	0.1 (50)	0.3	0.4 (10)	0.2 (−50)	0.2 (−50)	0.5 (50)
SDNND_b_	0.1	0.1	0.1 (20)	0.1 (0)	0.2 (40)	0.4	0.7 (80)	0.4 (10)	0.4 (10)	1.6 (300)

### 3.3. Experiment 1: relationships between isotopic and physiological responses

The normalized δ^15^N values were best explained by body mass, parasite infestation and VE% (Hypothesis 2; table 5). Higher infestation rate and larger body mass were associated with higher δ^15^N values, whereas VE% was negatively related to δ^15^N values, with ^15^N enrichment in females with a higher percentage of aberrant embryos. Body mass alone was the best predictor for the δ^13^C values (table 5).

**Table 5.** Most parsimonious linear models testing relationships between isotope composition and physiological status measured as reproductive performance (embryo viability, VE%), parasite infestation (Experiment 1) and growth/body condition indices, and survivorship and oxygen penetration depth (Experiment 2) in *M. affinis.* Greater oxygen depth indicates more oxygenated sediment. The isotope data were normalized to the treatment mean values to eliminate differences related to the baseline variability (see Material and methods for details).

dependent	explanatory variables	estimate	s.e.	*t*-value	*p*-value
experiment 1					
δ^15^N_a_	parasite infestation	0.177	0.084	2.100	0.039
	body mass	0.000	0.000	4.266	<0.001
	VE%	−0.206	0.104	−1.974	0.051
δ^13^C_a_	body mass	−0.000	0.000	−3.971	<0.001
experiment 2					
δ^15^N_a_	body mass	−1.065	0.232	−4.600	<0.001
δ^13^C_a_	amphipod mortality	0.060	0.031	2.233	0.030
	oxygen depth	0.050	0.022	1.941	0.058

### 3.4. Experiment 2: effects of feeding regime on growth performance and isotope composition

The median survival rate (45%) was similar among the treatments (Kruskal–Wallis: *H* = 0.731, *p* = 0.865; table 2). The weighted mean oxygen penetration depth (12.3 ± 2.1 mm) was not significantly different between the treatments (ANOVA: *F*_3,16 _= 0.591, *p* = 0.63) and did not correlate with the survival (*r* = 0.336, *p* = 0.147). Individual body mass in diatom and Tetraphyll treatments was significantly higher than for the initial animals, indicating positive growth during the experiment; in line with that, the amphipods in the diatom treatment also had a significantly higher RNA : DNA ratio than the initials (table 2, electronic supplementary material, table S5). Moreover, in the high-quality food treatments (diatoms and Tetraphyll), individuals were on average 20–25% larger, their RNA : DNA ratios were 20–24% higher, and the C : N ratios were 9–18% higher, compared to the reference animals (table 2). Accordingly, individual body mass, C : N ratio and RNA : DNA ratio were significantly higher in the high-quality food treatments than in the low-quality food (reference and lignin) treatments (electronic supplementary material, table S6). The variances of the C : N ratio and δ^13^C and δ^15^N values were significantly or marginally significantly lower in high-quality treatments than in the reference treatment (Levene's test; table 2). Variability in body condition and isotope data from all treatments are visualized in a PLS bagplot (figure 1*b*). The ED values were significantly lower in the animals supplied with high-quality food compared to those in the reference, whereas they did not differ from the reference in the lignin treatment (Hypothesis 1; table 3).

### 3.5. Experiment 2: effects of different feeding regimes on isotope niche metrics

Compared to the initial amphipods, those fed high-quality food had lower values for all niche metrics, except the SDNND. By contrast, all metrics were higher in the amphipods without supplemental food, i.e. reference and lignin treatments (Hypothesis 1, table 4, electronic supplementary material, figures S9–S14). The SEA_B_ values were significantly higher in the reference and lignin treatments compared to both diatoms and Tetraphyll treatments (*p* < 0.05 in all cases; figure 3*b*).

### 3.6. Experiment 2: relationships between isotopic and physiological responses

In high-quality food treatments, the growing animals approached the new diet, resulting in a negative relationship between the growth measured as a relative weight change and the corresponding δ^13^C change from the initial mean value (electronic supplementary material, table S7 and figure S1). In the lignin treatment, the relationship between the growth and the δ^13^C change was weakly positive (electronic supplementary material table S7 and figure S1), in concert with slow or no growth and no indication of lignin being used as a food. The relationship between the δ^15^N change and relative weight change was significantly negative in all treatments (electronic supplementary material figure S1 and table S7).

Variation in the normalized δ^15^N values was best explained by body mass (negatively), whereas the δ^13^C values were best explained by amphipod mortality and oxygen penetration depth (table 5, Hypothesis 2).

### 3.7. Combining physiological and isotopic measurements to identify stressed populations

The logistic regression identified ED and VE% as significant predictors of contaminant exposure with an odds ratio of 3.4 (table 6). The regression sensitivity and specificity were 60% and 70%, respectively (ROC analysis, AUC = 81%; electronic supplementary material, figure S2*a*). The ED and C : N ratio were the best predictors of the nutritional environment, with a model odds ratio (OR) of 38.5 (table 6). The regression sensitivity and specificity were 73% and 69%, respectively (ROC analysis, AUC = 92%; electronic supplementary material, figure S2*b*).

**Table 6.** Most parsimonious models for logistic regressions with treatment as a categorical response variable, i.e. exposure to contaminants in Experiment 1 and malnutrition in Experiment 2. For the malnutrition group, the data for animals receiving lignin or no food supplement were pooled. Compare the PLS bagplot for visualization of the results (figure 1).

predicted probability	predictors	estimate	s.e.	*z*-value	*p*-value	OR (unit change)
experiment 1						
probability of exposure to contaminants	ED	2.832	1.108	2.556	0.011	16.98
	VE%	−4.573	2.129	−2.148	0.032	0.010
experiment 2						
probability of malnutrition	ED	2.959	0.754	3.924	<0.001	19.27
	C : N	−0.689	0.497	−1.387	0.165	0.502

## 4. Discussion

Using controlled experiments with the amphipod *M. affinis*, we demonstrated that Layman niche metrics respond to environmental stress. The chemically or nutritionally stressed amphipods exhibited lower growth rate, body condition and reproductive performance, while also having a greater isotopic niche than the non-stressed animals. This apparent niche expansion was largely driven by animal growth, thus supporting the hypothesized associations between the isotope composition and growth. This is in line with the recently reported effects of individual growth rate on the isotope niche estimates in mysids receiving the same diet [19]. We show that other non-dietary variables, such as parasite infestation, embryo viability and oxygen conditions, also influenced isotope composition, and hence isotopic niche indices. The fact that isotope composition may reflect physiological status rather than dietary divergence has important implications for interpreting consumer isotope data in general and isotopic niche analysis in particular; previous work on isotope niche size might, in some cases, need to be reinterpreted and future studies should include measures of growth status when evaluating stable isotope data in consumers. However, variability in isotope composition among individuals could potentially be used as a diagnostic tool of environmental stress when combined with measures of physiological status. This is highly relevant for environmental assessment with isotope niche as a population-level response to environmental change.

### 4.1. Niche shifts and expansion under different feeding regimes

In agreement with Hypothesis 1, the amphipods fed high-quality food (commercial fish food and diatoms) had relatively narrow isotopic niches, suggesting diet similarity between the individuals of the same group. As expected, in these treatments, high growth was associated with the greater incorporation of added carbon (electronic supplementary material, figure S1), higher protein synthesis and body condition, compared to both initial amphipods and those in the reference treatment. The low-quality food treatments (reference and lignin) had no significant growth compared to the initials, and a weak positive relationship between growth and δ^13^C was observed in the lignin-exposed animals, supporting the view that animals exhibiting negative or near-zero growth may recycle ^12^C or rearrange fatty acids with concomitant effects on δ^13^C values [59]. It is important to note that none of the treatments represented starvation conditions, because the reference sediment was comparable to the nutritional conditions in the field during summer [60]. Starvation is known to increase δ^15^N values in consumers due to the loss of light isotopes in deamination [12,23]. In agreement with Hypothesis 2, the significant negative relationship between the amphipod body mass and δ^15^N values together with declining RNA : DNA ratios indicate that slow-growing individuals with low metabolic rates may recycle some endogenous nitrogen and have low protein synthetic activity, resulting in the enriched δ^15^N values. Moreover, higher δ^15^N values were observed in lignin-fed amphipods compared to the reference animals (table 2). It is possible that the layer of lignin, which is a highly refractory carbon source with only negligible amount of nitrogen (electronic supplementary material, table S2), reduced access of the animals to the underlying sediment, thus further limiting their nutrition. This may have facilitated nitrogen deficiency and a higher incidence of trans- and deamination, with subsequent enrichment in consumer δ^15^N values [61]. In mysids, a significant negative relationship between ^15^N fractionation and growth rate was demonstrated experimentally [19]. Similarly, Karlson *et al*. [62] found that the sediment–animal δ^15^N difference, a proxy for the trophic enrichment factor in benthic suspension- and deposit-feeders, was higher in habitats with nutrient-poor sediment. An alternative explanation to the negative relationship between the body size and δ^15^N values in the experimental animals is that the amphipods from the reference and lignin treatments were also feeding on meiofauna present in the sediment (mainly ostracods and nematodes in relatively low numbers, [34]) or decomposing dead amphipods. This type of feeding would not satisfy nutritional requirements and support adequate growth, yet result in a higher TP of the amphipods.

In the non-starved animals, the δ^13^C values were expected to be positively influenced by growth [19,26]. Indeed, the RNA : DNA ratio was positively correlated to δ^13^C values (not included in the best model in table 5, *p* = 0.08); however, sediment oxygen penetration depth and amphipod survival were the best predictors of δ^13^C. The most likely explanation is that more active amphipods oxygenate sediment via burrowing, respiring more ^12^C while retaining ^13^C. In the lignin and reference treatments, large NR, ‘trophic length of the population’, and CR, ‘diet diversification potential’ (*sensu* Layman *et al*. [2]), reflected not the trophic niche expansion compared to the initial population, but the physiological state of the animals in the nutritionally poorer conditions. Previous temporal field studies on *M. affinis* have found that the expansion of isotopic niche metrics following a settling bloom of mixed phytoplankton species may indeed reflect diet diversification [60], or alternatively, relatively poor nutritional conditions when compared with spring bloom input earlier in the year.

### 4.2. Niche expansion and metabolic depression in the contaminant-stressed population

When gravid amphipods from a relatively pristine area were exposed to the contaminated or non-contaminated sediments, both fecundity and embryo viability were significantly lower in the contaminated sediment confirming its toxicity (28% and less than 8% aberrant embryos in the exposure and reference, respectively; Experiment 1). In agreement with Hypothesis 1, the niche metrics of the amphipods exposed to the contaminated sediment were higher than in the reference sediment, even though amphipods in both treatments were supplied with the same, high-quality food in large quantities. The sediments used in the experiment were low in carbon and nitrogen content compared to the ambient sediment at the place of amphipod collection (e.g. C content of less than 2% in the experiment compared to 5% in the field; electronic supplementary material, tables S1 and S2). As the mean amphipod δ^15^N and δ^13^C values at the end of the experiment were closer to the signature of the added food than the sediment signal (compare table 1 and electronic supplementary material, table S1), it is unlikely that some alternative food sources like small heterotrophs and bacteria in the experimental sediment have contributed substantially to the amphipod diet and thus influenced their isotope composition, unless they were remarkably close to the isotopic signature of the experimental diet. We rather believe that the isotopic niche expansion (twofold) in the contaminant-stressed amphipods is related to the metabolic depression and detoxification. Increased ^15^N fractionation, as a result of intensified trans- and deamination during metabolic turnover involved in detoxification [13,14,63,64], was expected in the contaminant-stressed amphipods. Although both δ^15^N and δ^13^C values were influenced by body mass, the δ^15^N values were also partly explained by variation in embryo viability, supporting the link between reproductive toxicity and enriched δ^15^N values. Moreover, similar to the recently reported positive effect of microsporidian parasite infestation on the δ^15^N values in daphnids [15], we also observed this effect in the amphipods parasitized by nematodes. In agreement with this study, we did not observe any parasite effects on the δ^13^C values. Furthermore, the parasite infestation had a negative effect on fecundity, demonstrating the importance of including this parameter in ecotoxicological surveys. The lower fecundity in the infested females could be explained by the consumption of host lipids by the parasite, resulting in less resources for egg production, as shown, for example, in *Daphnia* infested by microsporidians [65]. Interestingly, the fecundity reduction was only evident in the reference treatment. This suggests that the infestation may partly counteract toxic stress by, for example, upregulation of the antioxidant defence system and heat shock protein response during the exposure [66,67] or that the nematodes themselves accumulated most of the ingested contaminants, thereby lowering body burdens of contaminants in the host [68]. Increased stress protein production [69], particularly under additional stress may be the cause of the observed enrichment in δ^15^N. The results suggest that parasite infestation is important to consider when interpreting isotope data and calculations of TP. In amphipods and other crustaceans, this might be challenging, particularly when infestation signs are not visible and cannot be recorded as presence/absence in regular surveys.

To sum up, alterations in nitrogen and carbon metabolism have probably contributed to the observed expansion of the niche metrics in the contaminant-exposed population, a pattern which was even more pronounced when including parasite-infested individuals in the calculations. The expansion in isotopic niche metrics for contaminant- and parasite-stressed amphipods were nearly as big as for malnutritioned amphipods, relative to respective experiment reference values. However, the absolute values for malnutritioned amphipods were larger. It is difficult to compare populations of *M. affinis* in different stages of their life cycle (investment in gonads during summer, while no somatic growth can be expected for gravid females in winter) and between years, where phytoplankton bloom intensity and hence growth responses can be expected to differ [60]. Testing the interacting effects of these stressors on isotope niche, under controlled conditions, would be very interesting.

### 4.3. Intra-population variability in isotope composition as an indicator of physiological stress

The combined results from our experiments support Hypotheses 1, 2 and 3; that greater isotopic niche indices, in particular the NR, could result from inter-individual variability in physiological status of consumers (phenotypic plasticity) rather than the diet diversification *per se*. Moreover, the intra-population variability in the δ^15^N and δ^13^C values combined with data on body condition could facilitate diagnostics of nutritionally stressed populations with high accuracy (92%). The model predicting contaminant exposure used variability in the δ^15^N and δ^13^C values (ED) combined with embryo viability and exhibited better specificity (the proportion of amphipods correctly identified as experiencing contaminant stress, 70%) than for sensitivity (60%), suggesting that additional biomarkers of physiological status could improve the prediction accuracy. Stressor-specific biomarkers of exposure can help in determining whether an amphipod has been exposed to natural or anthropogenic stress (e.g. contaminated sediment: [42,70]). They can also help in establishing cause–effect relationships, particularly when combined with ecologically relevant endpoints (such as embryo viability; e.g. [33,70]). In the context of environmental monitoring and ecological risk assessment, these relationships can be useful for detecting *in situ* stress on a population level. To improve measures of the trophic (isotopic) niche in invertebrates, particularly for those living in sediments where there are multiple potential food sources such as bacteria and fungi which cannot easily be extracted and analysed for SIA, molecular diet analysis could be used in combination with SIA [71]. Such an approach, combined with measures of growth status, could potentially separate a physiological stress effect from real diet diversification.

The fact that estimates of the TP and isotopic niche can be confounded by non-dietary parameters, including environmental stress, is of high relevance in environments chronically exposed to nutrient imbalance or anthropogenic pollutants. It is important to understand that true diet diversification cannot solely be assayed from isotope niche analyses without taking the physiological status of animals and environmental conditions into account. The apparent expansion of the isotopic niche resulting from stress-induced variability in physiological status can erroneously be interpreted as dietary divergence among individuals. We found that slow-growing amphipods had isotopic niche sizes several-fold larger than the fast-growing, fed on a high-quality food source. Contaminant-exposed and parasite-infested amphipods also nearly doubled the niche sizes compared to unstressed animals. We therefore recommend assessments of physiological status, including growth indices and parasite infestation, when interpreting consumer stable isotope data in general and isotopic niche metrics in particular. This study also demonstrates that the isotopic niche responds to environmental stress and could be used to study population-level responses to environmental change.

## Supplementary Material

Supplementary tables and figures

## Supplementary Material

The datasets supporting are uploaded at DRYAD and will be available if the manuscript is accepted (doi:10.5061/dryad.8h439).

## Data accessibility

The datasets supporting are available at the Dryad Digital Repository: http://dx.doi.org/10.5061/dryad.8h439 [72].
