# Supplementary material for: Isotopic niche reflects stress-induced variability in physiological status
Source: R Soc Open Sci. 2018 Feb 21;5(2):171398. doi: 10.1098/rsos.171398 (PMC5830748; doi:10.1098/rsos.171398)
Supplement: Supplementary tables and figures [file rsos171398supp1.docx]

**Supplementary Material**

**Isotopic niche reflects stress-induced variability in physiological status**

Agnes ML Karlson*^1,2^, Martin Reutgard^1^, Andrius Garbaras^3^, Elena Gorokhova ^1^

^1^Department of Environmental Science and Analytical Chemistry, Stockholm University, Svante Arrhenius väg 8, SE-106 91 Stockholm, Sweden

^2^Department of Ecology, Environment and Plant Science, Stockholm University, Svante Arrhenius väg 20, SE-106 91 Stockholm, Sweden

^3^Mass Spectrometry Laboratory, Centre for Physical Science and Technology, Savanoriu 231, LT-02300 Vilnius, Lithuania

*corresponding author: [agnes.karlson@su.se](mailto:agnes.karlson@su.se)

Current address:

Agnes ML Karlson: Department of Ecology, Environment and Plant Science, Stockholm University, Svante Arrhenius väg 20, SE-106 91 Stockholm, Sweden

**Table S1**. Sum of PAHS, PCB congeners, metals and chlorophenols in surface sediment (0-2 cm) as well as elemental carbon content, the C:N ratio and isotope composition. Details on the contaminant analyses can be found in Löf et al. [1]. In the reference sediment, all concentrations except arsenic (As) were within levels indicative of pristine sediments, whereas several metals and PAHS in the contaminated sediment were highly elevated.

|  | Control | Exposed |
| --- | --- | --- |
| Σ 23 PAH (ng/g dw) | 55 | 811 |
| Σ 12 PCB (pg/g dw) | 4 | 14 |
| Σ 10 Metals (μg/g dw) | 76 | 304 |
| Σ 12 Chlorophenols (ng/g dw) | 21 | 41 |
| Carbon content (%) | 0.5 | 1.8 |
| C:N ratio (weight: weight) | 9.2 | 10.2 |
| δ^15^N | 1.6 | 3.0 |
| δ^13^C | -24.9 | -23.6 |

[1] Löf M, Sundelin B, Bandh C, Gorokhova E. 2016 Embryo aberrations in the amphipod *Monoporeia affinis* as indicators of toxic pollutants in sediments: a field evaluation. *Ecol. Indic.* **60**, 18–30. (doi:10.1016/j.ecolind.2015.05.058)

**Table S2**. Overview of the reproductive variables analyzed in Experiment 1. Classification of the embryo aberrations follows Löf et al. [1] with some simplifications; the morphological changes are described in detail in Sundelin and Eriksson [2]. Only individual-specific endpoints (F and VE%) were included in the statistical analyses. See text for details.

| **Reproductive metric** | **Explanation** | **Variable derived** |
| --- | --- | --- |
| Fecundity | Number of embryos in the brood | Embryos per female (F) |
| Viable embryos | Number of healthy embryos in the brood | Number of viable embryos per female (VE) and percentage of viable embryos (VE%) |
| Embryos with arrested development | Embryo development halted before gastrulation | Percentage of arrested embryos |
| Malformed embryos | Embryos with shortened and irregular midgut, limbs or compound eye | Percentage of malformed embryos |
| Membrane-damaged embryos | Embryos with damaged egg membranes, enlarged egg or irregular egg shape | Percentage of membrane-damaged embryos |
| Dead broods | Dead eggs or decomposing embryos, visible as a dark fat residue in the brood. Individual eggs/embryos cannot be identified. | Percentage of females carrying dead or partially dead broods |

[1] Löf M, Sundelin B, Bandh C, Gorokhova E. 2016. Embryo aberrations in the amphipod *Monoporeia affinis* as indicators of toxic pollutants in sediments: a field evaluation. *Ecol. Indic.* **60**, 18–30. (doi:10.1016/j.ecolind.2015.05.058)

[2] Sundelin B, Eriksson AK. 1998. Malformations in embryos of the deposit-feeding amphipod *Monoporeia affinis* in the Baltic Sea. *Mar. Ecol. Prog. Ser.* **171**, 165–180.

**Table S3**. Overview of growth and body condition indices analysed in Experiment 2.

| **Variable** | **Analysed in** | **Rationale for inclusion** | **Reference for *M. affinis*** |
| --- | --- | --- | --- |
| Individual body mass | Whole body (mg dry weight) | Fecundity is positively related to body mass | Eriksson Wiklund and Sundelin 2004 |
| C:N ratio | Whole body | Positively related to lipid content, proxy for body condition | Lehtonen 1996 |
| RNA:DNA ratio | 6^th^ pereiopod | Protein synthetic capacity, proxy for growth | Gorokhova et al. 2010 |

**Eriksson Wiklund** AK, Sundelin B. 2004. Biomarker sensitivity to temperature and hypoxia - A seven year field study. *Mar. Ecol. Prog. Ser*. **274**, 209-214.

**Lehtonen** KK. 1996. Ecophysiology of the benthic amphipod *Monoporeia affinis* in an open-sea area of the northern Baltic Sea: seasonal variations in body composition, with bioenergetic considerations. *Mar. Ecol. Prog. Ser.* **143**, 87-98. (doi: 10.3354/meps274209)

**Gorokhova** E, Löf M, Halldorsson H, Lindström M, Elfwing T, Tjärnlund U, Sundelin B. 2010. Single and combined effects of hypoxia and contaminated sediments on the amphipod *Monoporeia affinis*. *Aquat. Toxicol.* **99**, 263-274. (doi:10.1016/j.aquatox.2010.05.005)

**Table S4**. Elemental composition, amino acid concentrations (total hydrolysable amino acids, THAA) and proportion of polyunsaturated fatty acids and eicosapentaenoic acid (EPA) in the sediment and different types of organic matter added to the sediment as a food source for amphipods in Experiment 2. Amino-acid and fatty-acid data are extracted from Nascimento et al. [1]. Isotope signature for the added material (diatoms, Tetraphyll and lignin) as well as the sediment is shown. Data are mean and SD (organic matter; n=3-5, amphipods; n=15).

|  | Sediment only | Tetraphyll | Diatom | Lignin |
| --- | --- | --- | --- | --- |
| C % | 4.8 ± 0.7 | 43.1 ± 1.7 | 27.8 ± 0.7 | 58.0 ± 2.3 |
| N % | 0.6 ± 0.0 | 8.2 ± 0.4 | 3.0 ± 0.3 | 0.2 ± 0.0 |
| P % | 0.2 ± 0.0 | 1.2 ± 0.0 | 0.4 ± 0.0 | * |
| THAA (mmol g dw^-1^) | * | 3.7 | 4.8 | * |
| PUFA (%) | * | 37.4 | 8.6 | * |
| EPA, 20:5ω3, (%) | * | 3.6 | 7.1 | * |
| δ^15^N | 5.0 ± 0.7 | 6.1 ± 0.3 | 4.2 ± 0.1 | * |
| δ^13^C | -23.0 ± 0.6 | -22.2 ± 1.0 | -27.1 ± 0.2 | -29.6 ± 3.0 |

*under analytical detection limit,

[1] Nascimento FJA, Karlson AML, Näslund J, Gorokhova E. 2009 Settling cyanobacterial blooms do not improve growth conditions for soft bottom meiofauna. *J. Exp. Mar.Biol. Ecol.* **368**, 138-146. (doi:10.1016/j.jembe.2008.09.014)

**Table S5**. Linear mixed effect models (LMM) testing treatment effect on body mass in Experiment 1 and 2: and body condition (c) and growth status (d) in Experiment 2. As a treatment variable, food supplements (diatoms, lignin, Tetraphyll and sediment only) were used; initial amphipod values was set as the reference value in the LMM. See also Table S6, where the reference treatment was set as reference value and initials were excluded from the analyses.

| **Variable** | **Estimate** | **SE** | **t-value** | **p-value** |
| --- | --- | --- | --- | --- |
| ***Experiment 1*** | | | | |
| 1. ***Body mass (ww)*** |  |  |  |  |
| *a1)* Reference | 0.04773 | 0.03762 | 1.269 | 0.207 |
| *a2)* Exposed | -0.01985 | 0.03455 | -0.575 | 0.567 |
| ***Experiment 2*** | | | | |
| ***(b) Body mass (dw)*** |  |  |  |  |
| diatoms | 0.3766 | 0.1773 | 2.124 | 0.0378 |
| Tetraphyll | 0.4382 | 0.1812 | 2.418 | 0.0186 |
| lignin | 0.1258 | 0.1910 | 0.659 | 0.5125 |
| control | 0.1385 | 0.1708 | 0.811 |  |
| 1. ***C:N ratio*** |  |  |  |  |
| diatoms | 0.7431 | 0.5362 | 1.386 | 0.2196 |
| Tetraphyll | 0.2095 | 0.5425 | 0.386 | 0.7135 |
| lignin | -0.6023 | 0.5498 | -1.096 | 0.3153 |
| control | -0.2993 | 0.5288 | -0.566 | 0.5947 |
| ***(d) RNA:DNA ratio*** |  |  |  |  |
| diatoms | 0.3594 | 0.1950 | 1.844 | 0.0707 |
| Tetraphyll | 0.3093 | 0.1983 | 1.560 | 0.1246 |
| lignin | -0.0037 | 0.2066 | -0.018 | 0.9858 |
| control | -0.0077 | 0.1896 | -0.041 | 0.9676 |

**Table S6**. Most parsimounous linear mixed effect models (LMM) and Generalized Linear mixed effect models (GLMM) testing treatment effect on the physiological status. As a treatment variable, exposure to contaminants in Experiment 1 and food supplements (diatoms, lignin and Tetraphyll) in Experiment 2 were used; reference treatment was set as the reference value in the LMM in both experiments. Body mass data was log-transformed and VE%-data was arcsine-transformed prior to analyses.

| **Variable** | **Estimate** | **SE** | **t-value** | **z-value** | **p-value** |
| --- | --- | --- | --- | --- | --- |
| ***Experiment 1*** | | | | | |
| 1. ***Fecundity*** |  |  |  |  |  |
| Exposure | -0.2416 | 0.0619 |  | -3.90 | <0.0001 |
| Parasite | -0.2913 | 0.0464 |  | -6.27 | <0.0001 |
| Exposure x Parasite | 0.34377 | 0.0650 |  | 5.29 | <0.0001 |
| 1. ***VE%*** |  |  |  |  |  |
| Exposure | -0.2931 | 0.0993 | -2.951 |  | 0.0118 |
| Fecundity | 0.0114 | 0.0024 | 4.835 |  | <0.0001 |
| 1. ***Body mass (ww)*** |  |  |  |  |  |
| Exposure | -138.70 | 61.36 | -2.26 |  | 0.0249 |
| 1. **C:N ratio** |  |  |  |  |  |
| Exposure | 0.0284 | 0.05326 | 0.533 |  | 0.596 |
| ***Experiment 2*** | | | | | |
| 1. ***Body mass (dw)*** |  |  |  |  |  |
| diatoms | 0.0863 | 0.0639 | 1.351 |  | 0.183 |
| tetraphyll | 0.0979 | 0.0625 | 1.566 |  | 0.124 |
| lignin | -0.0284 | 0.0673 | -0.422 |  | 0.674 |
| diatoms+Tetraphyll* | 0.1038 | 0.0458 | 2.265 |  | 0.028 |
| 1. ***C:N ratio*** |  |  |  |  |  |
| diatoms | 1.0426 | 0.3863 | 2.699 |  | 0.0178 |
| tetraphyll | 0.5085 | 0.3950 | 1.287 |  | 0.2191 |
| lignin | -0.3047 | 0.4048 | -0.753 |  | 0.4628 |
| 1. ***RNA:DNA ratio*** |  |  |  |  |  |
| diatoms | 0.3672 | 0.1550 | 2.369 |  | 0.0222 |
| tetraphyll | 0.3170 | 0.1584 | 2.001 |  | 0.0514 |
| lignin | -0.0055 | 0.1719 | -0.032 |  | 0.9745 |

*as the reference treatment merged control and lignin data were used (see methods).

**Table S7.** Generalized linear models testing effects of treatment and relative growth rate on shifts in isotope values of amphipods (absolute change in δ^15^N and δ^13^N) in Experiment 2. As a treatment variable, organic matter supplements (diatoms, lignin and Tetraphyll) was used; the reference treatment was set as reference value in the GLM for δ^15^N_shift_. For δ^13^C_shift_ separate models were tested for high quality nutritional environments (diatom and Tetraphyll) and for lignin (see figure S1A and C for interaction between treatment and biomass change). Data was box-cox transformed prior to analyses to improve normality of residuals.

| **Variable** | **Estimate** | **SE** | **t-value** | **p-value** |
| --- | --- | --- | --- | --- |
| **δ^15^N_shift_** | | | | |
| diatoms | 0.5614 | 0.1528 | 3.673 | <0.001 |
| Tetraphyll | 0.4322 | 0.1566 | 2.758 | 0.0084 |
| lignin | 0.4916 | 0.1618 | 3.038 | 0.0040 |
| relative biomass gain | -0.0528 | 0.0093 | -5.690 | <0.001 |
| **δ^13^C_shift_** (high nutritional quality) | | | | |
| Tetraphyll | 3.8568 | 3.3681 | 1.145 | 0.0372 |
| relative biomass gain | -0.7306 | 0.3295 | -2.217 | 0.0372 |
| **δ^13^C_shift_** (lignin) | | | | |
| relative biomass gain | 0.03978 | 0.0213 | 1.864 | 0.0994* |

*significant in Spearman rank correlation.

**Supplementary figures**

**Figure S1.** Absolute change in δ^13^C (A, C, and E panels) and δ^15^N (B, D, and F panels) in relation to the relative change (%) in individual mass during Experiment 2 (δ^13^C-values for amphipods are lipid-corrected). The thin lines crossing at 0:0 indicate no change for each variable. The difference in the diet relative to the sediment is shown as a horizontal arrow. As lignin contains no nitrogen, there is no line indicating a shift from the sediment δ^15^N for lignin diet (D). Note the differences in the scale for the Y-axis within a variable.

-6

**A**

**B**

**Figure S2.** Receiver operating characteristic curve for GLM of Experiment 1 (A) and Experiment 2 (B). See Results section for the details and Table 4 for statistical output of logistic regression models, AUC, sensitivity and specificity of the models.

**Figure S3.** Density plots of the nitrogen range (NR) in initial animals and in animals from the references sediment, the contaminated sediment (exposed) and in parasitized animals in Experiment 1. Boxed areas indicates the 50, 75 and 95% credible interval (see text for details).

**Figure S4**. Density plots of the carbon range (CR) in initial animals and in animals from the references sediment, the contaminated sediment (exposed) and in parasitized animals in Experiment 1. Boxed areas indicates the 50, 75 and 95% credible interval (see text for details).

**Figure S5**. Density plots of the distance to centroid (CD) in initial animals and in animals from the references sediment, the contaminated sediment (exposed) and in parasitized animals in Experiment 1. Boxed areas indicates the 50, 75 and 95% credible interval (see text for details).

**Figure S6**. Density plots of the convex hull total area (TA) in initial animals and in animals from the references sediment, the contaminated sediment (exposed) and in parasitized animals in Experiment 1. Boxed areas indicates the 50, 75 and 95% credible interval (see text for details).

**Figure S7**. Density plots of the mean nearest neighbor distance (MNND) in initial animals and in animals from the references sediment, the contaminated sediment (exposed) and in parasitized animals in Experiment 1. Boxed areas indicates the 50, 75 and 95% credible interval (see text for details).

**Figure S8**. Density plots of the standard deviation of the nearest neighbor distance (SDNND) in initial animals and in animals from the references sediment, the contaminated sediment (exposed) and in parasitized animals in Experiment 1. Boxed areas indicates the 50, 75 and 95% credible interval (see text for details).

**Figure S9**. Density plots of the nitrogen range (NR) in initial animals and the four treatments in Experiment 2. Boxed areas indicates the 50, 75 and 95% credible interval (see text for details). Control denotes reference sediment.

**Figure S10**. Density plots of the carbon range (CR) in initial animals and the four treatments in Experiment 2. Boxed areas indicates the 50, 75 and 95% credible interval (see text for details). Control denotes reference sediment.

**Figure S11**. Density plots of the distance to centroid (CD) in initial animals and the four treatments in Experiment 2. Boxed areas indicates the 50, 75 and 95% credible interval (see text for details). Control denotes reference sediment.

**Figure S12**. Density plots of the convex hull total area (TA) in initial animals and the four treatments in Experiment 2. Boxed areas indicates the 50, 75 and 95% credible interval (see text for details). Control denotes reference sediment.

**Figure S13**. Density plots of the mean nearest neighbor distance (MNND) in initial animals and the four treatments in Experiment 2. Boxed areas indicates the 50, 75 and 95% credible interval (see text for details). Control denotes reference sediment.

**Figure S14**. Density plots of the standard deviation of nearest neighbor distance (SDNND) in initial animals and the four treatments in Experiment 2. Boxed areas indicates the 50, 75 and 95% credible interval (see text for details). Control denotes reference sediment.
